# Deoxynortryptoquivaline: A unique antiprostate cancer agent

**DOI:** 10.32604/or.2023.030266

**Published:** 2023-09-15

**Authors:** YOHKO YAMAZAKI, MANABU KAWADA, ISAO MOMOSE

**Affiliations:** 1 Institute of Microbial Chemistry (BIKAKEN), Numazu Branch, Microbial Chemistry Research Foundation, Numazu, 410-0301, Japan; 2 Institute of Microbial Chemistry (BIKAKEN), Laboratory of Oncology, Microbial Chemistry Research Foundation, Shinagawa-ku, 141-0021, Japan

**Keywords:** Anticancer antibiotics, Prostate cancer, Androgen receptor, Androgen dependency

## Abstract

The androgen receptor (AR) is a critical target in all the clinical stages of prostate cancer. To identify a new AR inhibitor, we constructed a new screening system using the androgen-dependent growth of prostate cancer cell lines as a screening indicator. We screened 50,000 culture broths of microorganisms using this screening system and found that the fermentation broth produced by a fungus inhibited androgen-dependent growth of human prostate cancer LNCaP cells without cytotoxicity. Purification of this culture medium was performed, and this resulted in deoxynortryptoquivaline (DNT) being identified as a novel inhibitor of AR function. DNT showed potent inhibition of androgen-dependent growth of human prostate cancer LNCaP cells. The AR competitor assay was performed, and DNT did not act as an AR antagonist. However, DNT inhibited AR-dependent transcriptional activity and AR nuclear translocation, it suggested that the suppression of AR function leads to inhibition activity against androgen-dependent growth.

## Introduction

The estimated cancer incidence in Japan in 2021 was approximately 1,009,800 of which 95,400 was prostate cancer and this was the most common male cancer site [1]. Prostate cancer is initially an androgen-dependent tumor and androgen deprivation therapy (ADT) is effective. However, despite an initial remission with ADT, prostate cancer progresses to an androgen-independent stage and acquires resistance to androgen deprivation therapy, and develops castration-resistant prostate cancer (CRPC). CRPC does not respond to standard cytotoxic chemotherapy, and the prognosis is not good.

Several molecular mechanisms are known to enhance the progression of prostate cancer to CRPC, including androgen receptor (AR) gene mutation, amplification, or overexpression [2–4], post-translational modifications, co-activator, and co-repressor modifications [5], alternative pathways bypassing AR signaling [6], and ligand-independent activation of AR [7]. In addition, AR splice variants have recently been identified which are constitutively active because they lack the ligand-binding domain [8–11].

Prior to 2010, docetaxel was the first and only life-prolonging agent available for metastatic CRPC [12]. In the last few years, second-generation AR antagonists for CRPC have been approved for clinical use and three new drugs, enzalutamide [13–15], apalutamide [16,17], and darolutamide [18–21], have been shown to extend survival in men with CRPC.

The efficacy of these new drugs proves that AR is a principal driver of prostate cancer progression in patients with CRPC. However, these agents only provided a temporary response, because resistance is rapidly developed [22,23]. We hypothesized that a new type of AR inhibitor, other than AR antagonists, could serve as a unique therapeutic agent for prostate cancer. Microbial secondary metabolites have been recognized as a primary source of new compounds for drug discovery, and thus we selected these as a screening source. Here we report the construction of a screening system for an AR inhibitor, and the identification of deoxynortryptoquivaline (DNT) through a screening study and its biological activity.

## Materials and Methods

### Cell culture

Human prostate cancer cell lines LNCaP, VCaP, and 22Rv1 were purchased from ATCC (Manassas, VA, USA). LNCaP and 22Rv1cells were grown in RPMI 1640 medium (NISSUI, Tokyo, Japan) containing 10% FBS (MP Biomedicals, Strasbourg, France), 0.3 mg/ml glutamine. VCaP cells were grown in Dulbecco’s modified Eagle medium (NISSUI) containing 10% FBS (Sigma-Aldrich, St Louis, MO, USA).

### Sera

The 18 serums used were provided as shown below. No.1–5 FBS (Sigma-Aldrich, St Louis, MO, USA), No.6–7 FBS (Thermo Fisher Scientific, MA, USA), No.8–9 FBS (Biowest, Nuaille, France), No.10–11 FBS (Biological Industries, Beit Haemek, Israel), No.12 FBS (Cytiva, Tokyo, Japan), No.13–14 FBS (VERITAS, Tokyo, Japan), No.15–18 FBS (MP Biomedicals, Strasbourg, France).

### Determination of androgen-dependent growth of prostate cancer cells

For the androgen-dependent assays, LNCaP and VCaP cells were grown in phenol red-free RPMI 1640 medium (Life Technologies, CA, USA) containing 2% charcoal-stripped FBS (cFBS) for 1 day and treated with the sample to be measured or 1 nM R1881 for 5 days. For the androgen-independent (cytotoxicity) assays, LNCaP and VCaP cells were grown in phenol red-free RPMI 1640 medium containing 2% cFBS for 1 day and treated with the sample to be measured and 10% FBS No.18 for 5 days. Cell viability was determined using an MTT (3-[4,5-dimethylthiazol-2-yl]-2,5-diphenyltetrazolium bromide) assay and shown as percentage of untreated controls.

### Screening for AR inhibitors

We screened 50,000 culture broths of microorganisms using the constructed screening system. Because the inhibition of androgen-independent growth is an indication of cytotoxicity, a compound which inhibits only androgen-dependent growth is a candidate. The screening was performed using only LNCaP cells.

### RNA isolation and real-time RT-PCR

LNCaP and VCaP cells were cultured in phenol red-free RPMI 1640 medium supplemented with 2% cFBS for 1 day and treated with 2% or 10% FBS No.18 and 1 nM R1881 for 24 or 48 h. Total RNA was isolated using the RNeasy Mini Kit (Qiagen, Hilden, Germany) according to the manufacturer’s protocol. cDNA was synthesized from 1 µg RNA using the Promega Reverse Transcription System (Promega, Madison, WI, USA). Real-time RT-PCR was performed on a Thermal Cycler Dice Real Time System (Takara, Shiga, Japan) using SYBR® Premix Ex Taq™ II (Takara). All primers used were purchased from Takara. The following primers were used: PSA-forward: 5′-ATGTGGGTCCCGGTTGTCTT-3′ and PSA-reverse: 5′-CCACAATCCGAGACAGGATGAG-3′, KLK2-forward: 5′-CGAAGACTGGCAACTTGGCTTTA-3′, KLK2-reverse: 5′-ATCAGCTACACTCCACAAGGTCCTC-3′, TMPRSS2-forward: 5′-CCTGGATGGTGGCCAGAAATA-3′, TMPRSS2-reverse: 5′-CGCACCAAGGGCACTGTCTA-3′, β-actin-forward: 5′-TGGCACCCAGCACAATGAA-3′, β-actin-reverse: 5′-CTAAGTCATAGTCCGCCTAGAAGCA-3′. All reactions were run in triplicate, and the values were normalized to the level of β-actin as a reference. Data are representative of at least 3 independent experiments.

### In vitro ligand-binding assay

AR competition assays were performed using the PolarScreen AR Competitor Assay kit (Thermo Fisher Scientific, Waltham, MA) and carried out as described previously [24]. Data are representative of at least 3 independent experiments.

### Western blot analysis

LNCaP and VCaP cells were cultured in phenol red-free RPMI 1640 medium supplemented with 2% cFBS for 1 day and treated with 1.0 µg/mL DNT or 1 nM R1881 for the indicated durations. Preparation of cell lysate and western blot analysis was carried out as described previously [24]. The primary antibodies used included anti-AR (sc816, Santa Cruz, Dallas, TX, USA), anti-PSA (ab76113, Abcam, Cambridge, UK), anti-Cyclin D1 (sc-246, Santa Cruz), anti-nucleolin (sc-13057, Santa Cruz), and anti-tubulin (T5168, Sigma-Aldrich).

The fractionation of LNCaP cells into cytosolic and nuclear fractions was performed using a nuclear/cytosol fractionation kit (BioVision Inc., Mountain View, CA) according to the manufacturer’s instructions. Digitalization was performed with ImageJ.

### Transfection and dual luciferase activity assay

LNCaP, VCaP, and 22Rv1 cells were cultured in phenol red-free RPMI 1640 supplemented with 10% FBS in 96-well plates. After incubation for 24 h, transfection for dual luciferase assay was carried out as described previously [24]. 1 nM R1881 and DNT at each concentration were added to the cells and luciferase activity was measured after 24 h using the Dual-Glo luciferase reporter assay kit (Promega).

### Isolation of DNT

The fermentation broth CR17072 (8000 mL) was centrifuged and the fungus body was removed. The broth was extracted with ethyl acetate and added to a silica gel 60 (Merck KGaA, Darmstadt, Germany) and the active fractions were eluted with chloroform: methanol (10:1, v/v). Next, the active fractions were applied to a Sephadex LH-20 column (GE Healthcare, Pittsburgh, USA) and eluted with methanol. The active fractions were further purified by HPLC (Capcell Pak C18 UG, Shiseido, Tokyo, Japan, 20 × 250 mm; solvent, gradient elution with a mixture of water/acetonitrile, UV at 250 nm; flow rate, 10 mL/min). DNT and nine types of tryptoquivalines were obtained from a similar UV spectrum. The fractions were further concentrated under reduced pressure to provide pure compounds as white powders.

### Immunostaining

pTriAR plasmid expressing full length AR was provided by Taiho Pharmaceutical. DU145 Cells were transfected with pTriAR plasmid using FuGENE HD transfection reagent according to the manufacturer’s protocol.

LNCaP and DU145 Cells with stably transfected AR were grown on glass coverslips, then fixed with cold acetone for 2 min at room temperature. Following fixation, cells were blocked with FBS for 20 min and incubated with AR antibodies (sc816, Santa Cruz) for 60 min. The appropriate secondary antibodies conjugated with Alexa 488 (green) (Cell Signaling Technology, Beverly, MA, USA) were added and allowed to incubate for 45 min. Cells were counterstained with propidium iodide for 10 min and visualized by fluorescence microscopy.

## Results

### Construction of the screening system

Early-stage prostate cancer requires androgens for growth and maintenance like normal prostate tissue, of which both are AR-dependent. On the other hand, the androgen-independent pathway is acquired after endocrine therapy and contributes to endocrine therapy resistance.

Recently, new-generation hormonal agents have shown efficacy in improving the overall survival, proving the hypothesis that CRPC remains dependent on the androgen signaling axis. AR antagonists are currently the only clinical drugs that function as AR inhibitors. Therefore, we hypothesized that a new type of AR inhibitor could serve as a unique therapeutic agent for prostate cancer. We selected the androgen-dependent growth of prostate cancer cell lines as a screening indicator because it can detect multiple steps of AR signaling. Thus, we needed to differentiate androgen-dependent from androgen-independent growth in prostate cancer cells.

**FIGURE 1:**
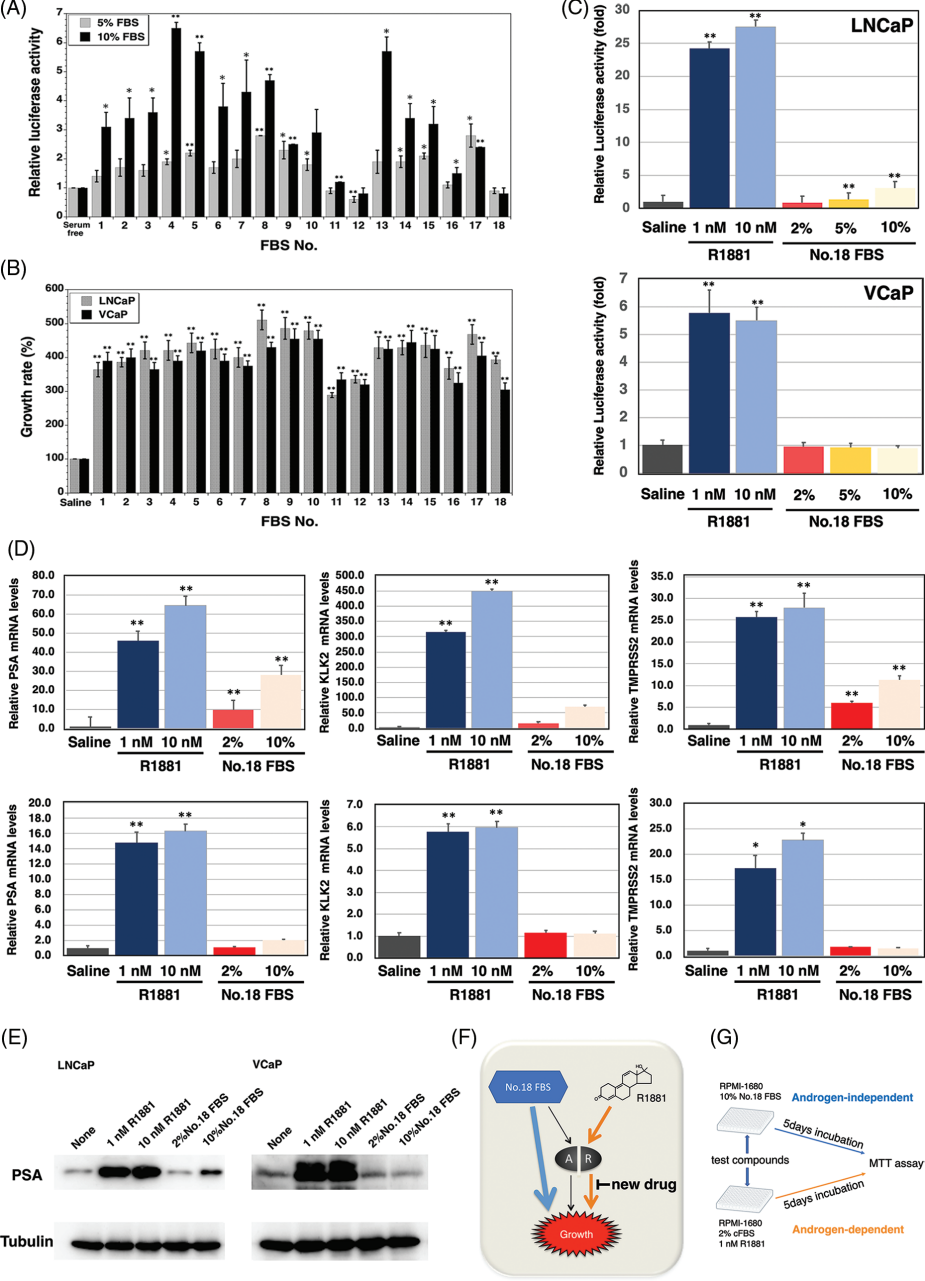
Construction of the screening system for the inhibitor of androgen-dependent growth in prostate cancer cells. (A) Androgen receptor (AR) transcriptional activity of 18 fetal bovine serums (FBSs) by the dual luciferase reporter system (*p*-value: ** < 0.01, * < 0.05 in comparison with saline-treated controls) (B) The growth of the androgen-sensitive LNCaP and VCaP prostate cancer cells stimulated with the 18 FBSs (*p*-value: ** < 0.01 in comparison with saline-treated controls) (C) AR transcriptional activity of R1881 and FBS No.18 by the dual luciferase reporter system (*p*-value: ** < 0.01 in comparison with saline-treated controls) (D) PSA, TMPRSS2, and KLK2 mRNA levels of LNCaP and VCaP prostate cancer cells stimulated with R1881 and FBS No.18 (*p*-value: ** < 0.01, * < 0.05 in comparison with saline-treated controls) (E) PSA protein levels of LNCaP and VCaP prostate cancer cells stimulated with R1881 and FBS No.18. (F) AR-dependent and AR-independent growth in LNCaP and VCaP prostate cancer cells stimulated with R1881 and FBS No.18. (G) Schematic representation of the screening method.

The specificity of AR ligand frequently becomes broadened in advanced prostate cancer indicating that AR can be activated by nonandrogenic molecules including androgen antagonists, corticosteroids, and 17-estradiol. Accordingly, we researched the amount of AR ligands in sera using a reporter assay. The 18 types of sera enhanced the AR-dependent transcriptional activity with varying intensities, and it was suggested that these 18 sera contained different amounts of AR ligands (Fig. 1A). In addition, we checked the growth of LNCaP and VCaP prostate cancer cells with the various sera, so that LNCaP and VCaP prostate cancer cells can be propagated by the sera, number 11, 12, and 18, and did not affect the AR-dependent transcriptional activity (Fig. 1B). As a result, we selected serum 18 because it had the lowest amount of AR ligand among the sera screened, and both prostate cancer cell lines were able to be propagated in the medium containing this serum by the androgen-independent pathway.

Next, we compared the transcriptional activities of the ligands of serum 18 and synthetic androgen methyltrienolone/R1881 (Fig. 1C). As expected, there was a 24- and 5.9-fold increase in AR transcript levels with 1 nM R1881 in LNCaP and VCaP cells, respectively. However, there was slight induction of AR-dependent transcriptional activity when LNCaP and VCaP cells were supplemented with 2%, 5%, and 10% of serum 18. In addition, we examined the effect of serum 18 and R1881 on the expression of androgen-regulated genes PSA, KLK2, and TMPRSS2 (Fig. 1D). Real-time PCR showed a significant induction of PSA, TMPRSS2, and KLK2 mRNA expression with the supplementation of 1 and 10 nM R1881 in LNCaP and VCaP prostate cancer cells. However, in LNCaP cells, 10% serum 18 provoked a lower induction of PSA, TMPRSS2, and KLK2 mRNA expression compared to R1881, and induction of three genes by 2% No.18 serum was even smaller. Additionally, there was no significant induction of the three genes in VCaP cells when supplemented with 2%, 5%, or 10% serum 18. Finally, we determined the induction of PSA protein expression by serum 18. Western blot analysis showed that R1881 induced a strong induction of PSA protein expression, and in contrast 2% serum 18 did not induce PSA protein expression in both prostate cancer cell lines (Fig. 1E).

As a result, androgen-dependent and androgen-independent pathways could be identified using R1881 and serum 18 in LNCaP and VCaP cell lines (Fig. 1F). Thus, we constructed a screening system for a new type anti-androgen as shown in Fig. 1G.

### Identification of DNT

To identify a new AR inhibitor, we screened 50,000 culture broths of microorganisms using the androgen-dependent growth of the human prostate cancer cell line LNCaP as a screening indicator. Furthermore, because the pathway for androgen-independent growth is also used by normal cells, the inhibition of androgen-independent growth leads to side effects. For this reason, the broth which inhibits both growths by No.18 FBS and R1881 are not a candidate.

**FIGURE 2:**
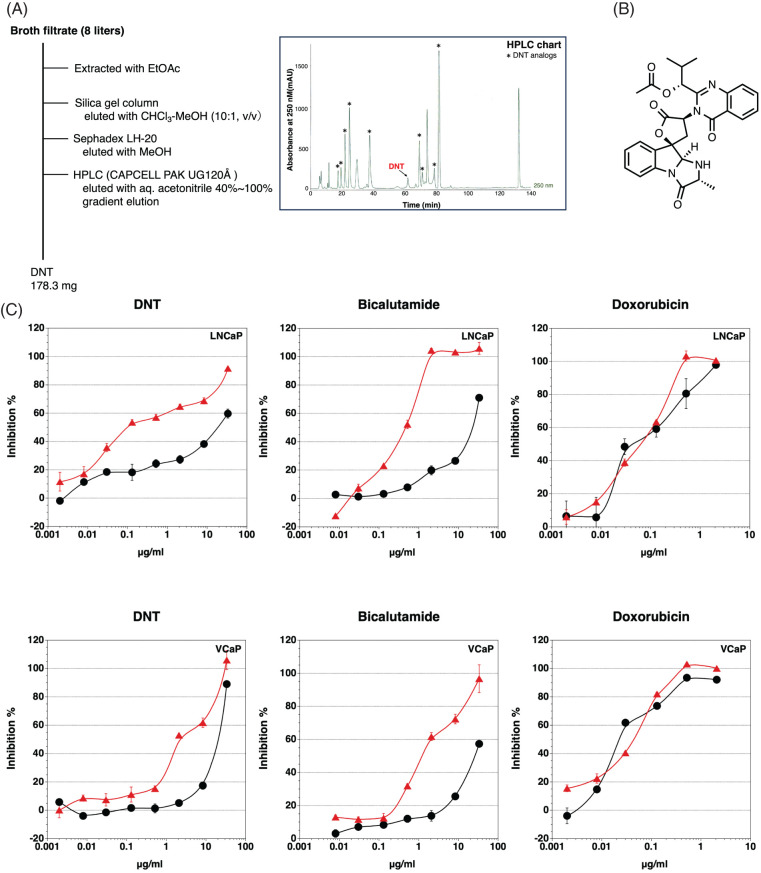
Identification of deoxynortryptoquivaline (DNT). (A) Schematic representation of the isolation of DNT from the fermentation broth CR17072 produced by a fungus. (B) The structure of DNT. (C) Inhibition of androgen-dependent growth of LNCaP and VCaP prostate cancer cells by DNT, bicalutamide, and doxorubicin. •, Cytotoxicity; 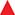, Inhibition of androgen-dependent growth. Data represent means ± SD of at least three independent experiments.

We found that the fermentation broth CR17072 produced by a fungus inhibited androgen-dependent growth without the inhibition of androgen-independent growth. The isolation of the inhibitor of androgen-dependent growth of LNCaP cells was performed using an ethyl acetate extraction method, silica gel chromatography, Sephadex LH-20 column, and High-performance liquid chromatography (HPLC) as shown in Fig. 2A. The structure determination was performed using high resolution electrospray ionization mass spectroscopy (HRESI-MS), nuclear magnetic resonance. The active compound in the CR17072 fermentation broth was determined to be deoxynortryptoquivaline (DNT) (Fig. 2B). DNT was found to as a mycotoxin [25] and has antiviral activity against influenza virus A (H1N1) [26].

DNT showed potent inhibition of the androgen-dependent growth of LNCaP cells, with low cytotoxicity. The inhibitory effect on the androgen-dependent growth was reduced in the VCaP cells compared to the LNCaP cells (Fig. 2C). In this assay, bicalutamide (a clinically used AR antagonist) showed similar specific cytotoxicity against the androgen-dependent growth of LNCaP and VCaP cells. Contrary to this, there was no difference between androgen-dependent proliferation and cytotoxicity in doxorubicin (DNA intercalator). Moreover, DNT did not show cytotoxicity against AR-negative cancer cell lines PC-3 and DU145 stimulated with No.18 serum (data not shown). There are 10 DNT analogs in the fermentation broth CR17072, but only DNT and deoxytoquivaline showed an inhibitory effect on the androgen-dependent growth of LNCaP. Because deoxytoquivaline showed cytotoxicity (inhibitory effect on androgen-independent growth), deoxytoquivaline was not considered as a candidate.

### Effect of DNT on AR signaling in prostate cancer cells

First, we performed the PolarScreen™ AR competitor assay to assess whether DNT acts as an antagonist. In this assay, AR [AR-LBD (His-GST)] forms a complex with its fluorescence-tagged ligand, resulting in a high polarization value. Competitors displace the fluorescently tagged ligand from the AR/ligand complex, resulting in reduced polarization values. DNT did not displace the fluorescent ligand from the complex and showed a high polarization value. By contrast, dihydrotestosterone (DHT, a potent AR agonist) and bicalutamide showed low fluorescence polarization with IC50 values of 1.1 and 490 nM, respectively (Table 1). This result indicates that DNT does not have affinity for AR-LBD and does not function as an AR antagonist, and DHT and bicalutamide have strong affinities for AR-LBD.

**TABLE 1:** Competitive effect of deoxynortryptoquivaline (DNT) on androgen receptor (AR). The competitive binding ability of DNT, dihydrotestosterone (DHT), and bicalutamide against AR-LBD, as evaluated by an AR fluorescence polarization assay

Ligand competition binding assay for AR	
Compound	IC_50_ (nM)
DNT	>500000
DHT	1.1
Bicalutamide	490

**FIGURE 3:**
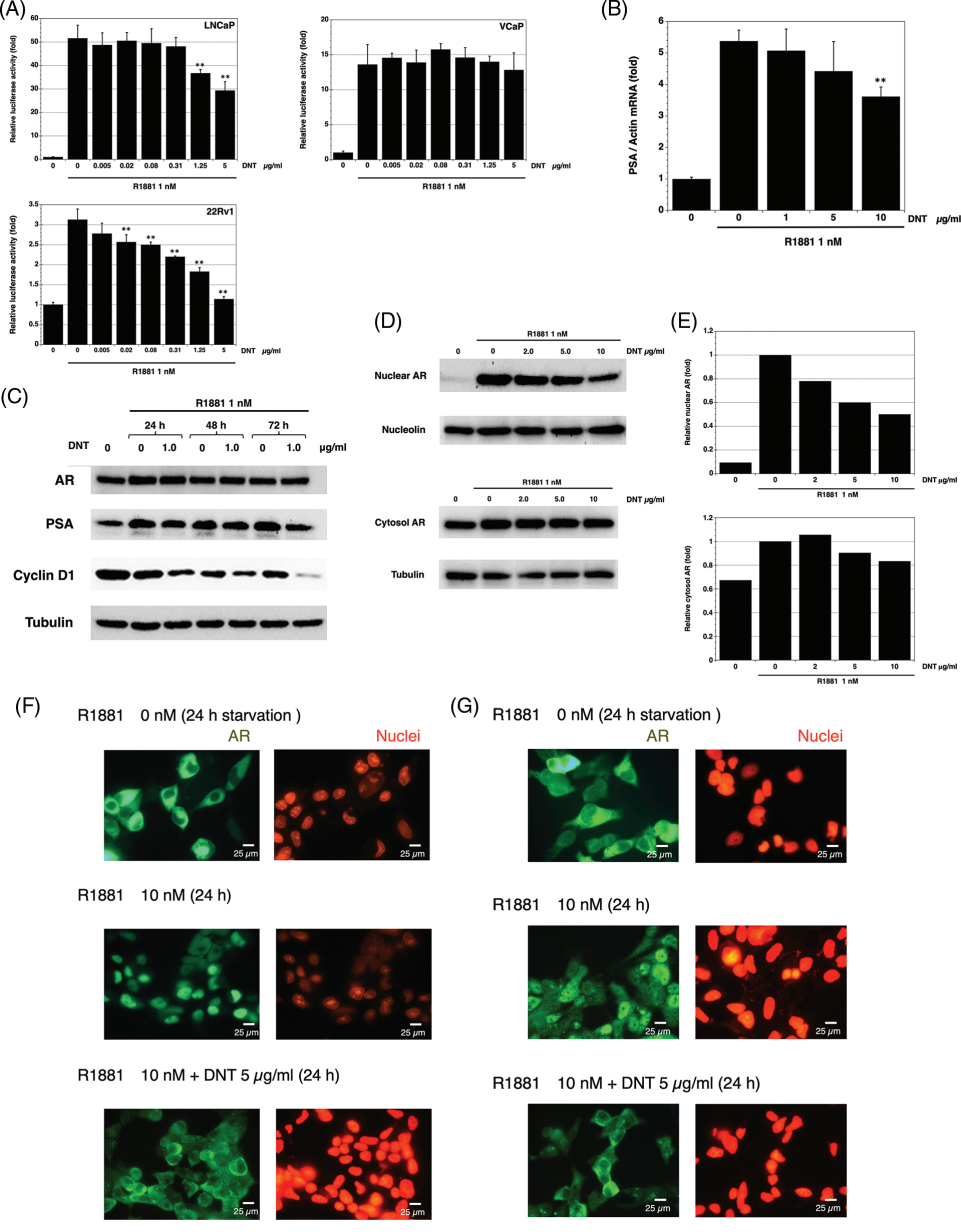
Effect of DNT on AR signaling in prostate cancer cells. (A) The inhibition effects of DNT on AR transcriptional activity in LNCaP, VCaP, and 22Rv1 prostate cancer cells. Data represent means ± standard deviation (SD) of at least three independent experiments (*p*-value: ** < 0.01 in comparison with saline-treated controls) (B) The inhibition effects of DNT on PSA mRNA levels in LNCaP prostate cancer cells. Data represent means ± standard deviation (SD) of at least three independent experiments (*p*-value: ** < 0.01 in comparison with saline-treated controls) (C) The effects of DNT on AR, PSA, and cyclin D1 protein levels in LNCaP prostate cancer cells. (D) The effects of DNT on nuclear and cytosol AR protein levels in LNCaP prostate cancer cells. AR protein levels were analyzed in LNCaP prostate cancer cells after 6 h of stimulation with R1881. (E) Western blot quantification was performed using ImageJ software (NIH). (F) Effects of DNT on AR nuclear localization in LNCaP prostate cancer cells. Scale bars represent 25 μm. (G) Effects of DNT on AR nuclear localization in DU145 cells with stably transfected AR prostate cancer cells. LNCaP and DU145 cells were fixed and immunostained with anti-AR antibody or propidium iodide. The green fluorescent staining indicates the AR, and red fluorescent staining indicates the nuclear. Scale bars represent 25 μm.

Next, we researched the effect of DNT on AR transcriptional activity using a dual luciferase reporter system. In this assay, all three human prostate cancer cell lines LNCaP, VCaP, and 22Rv1 cells showed an induction of luciferase activity with R1881. DNT inhibited the AR transcriptional activity in a dose-dependent manner in LNCaP, VCaP, and 22Rv1 cells, but the inhibitory effect on VCaP was slight (Fig. 3A).

Next, we investigated the effect of DNT on the expression of PSA mRNA expression in LNCaP cells. DNT repressed R1881-induced expression of PSA mRNA at 24 h of treatment (Fig. 3B).

Moreover, we investigated the effect of DNT on the expression of AR and PSA using western blotting in LNCaP cells. DNT did not affect the expression of AR protein and slightly reduced the expression of PSA protein. We determined the change of cell-cycle proteins and found that DNT repressed the expression of the cyclin D1 protein (Fig. 3C).

Finally, we investigated the effect of DNT on the AR nuclear translocation in LNCaP cells. Western blotting showed that R1881 induced and DNT repressed AR nuclear translocation in LNCaP cells (Figs. 3D and 3E). In addition, the inhibition of DNT on AR nuclear translocation induced by R1881 in LNCaP (Fig. 3F) and DU145 cells with stably transfected AR (Fig. 3G) was confirmed by image analysis of AR immunostaining.

## Discussion

AR is a principal driver of prostate cancer progression and an important therapeutic target for all clinical stages of prostate cancer. AR is activated by binding to androgens, dissociating from heat shock proteins and chaperones, dimerizing, and translocating into the nucleus. It then binds to the androgen response elements in the regulatory region of AR-dependent genes and activates the androgen signaling pathway [27].

Although second-generation AR antagonists have improved the overall survival of patients with prostate cancer, both androgen-dependent and CRPC, but potential side effects have been reported. Enzalutamide can cross the blood–brain barrier and antagonize the GABAα receptor in the central nervous system (CNS), and thus it is associated with CNS-related events such as seizure and falls [28–32]. The recent meta-analysis with regards to patients with CRPC that were treated with enzalutamide, darolutamide, or apalutamide, clarified that second-generation AR antagonists are associated with a significantly increased risk of cardiovascular events including stroke, heart failure, and peripheral vascular disease [33]. Furthermore, second-generation AR antagonists cannot completely cure patients with CRPC and ultimately the disease progresses to lethal neuroendocrine prostate cancer (NEPC). The incidence of NEPC has significantly increased with the clinical use of AR inhibitors [34–36]. For this reason, we hypothesized that a new type of AR inhibitor, other than AR antagonists, could serve as a unique therapeutic agent for prostate cancer.

We selected the androgen-dependent growth of a prostate cancer cell line as a screening indicator because androgen-dependent growth reflects the androgen activity and can detect multiple AR signaling steps. Human androgen-sensitive cells, LNCaP and VCaP, can grow with R1881 stimulation, and androgen-dependent growth was detected using LNCaP and VCaP cells. Because the inhibition of androgen-independent growth is an indication of cytotoxicity, a compound which inhibits only androgen-dependent growth is a candidate. Consequently, androgen-dependent and androgen-independent growth of LNCaP and VCaP cells needed to be differentiated. However, the common medium FBS contains androgens or AR ligands and activates the androgen signaling pathway. Because of this, 18 different sera were screened using a reporter assay to identify an FBS that does not activate the androgen pathway and it was found that LNCaP and VCaP cells grew in serum 18, which slightly activated the androgen pathway (Fig. 1). Moreover, serum 18 only slightly induces AR-dependent transcriptional activity or the androgen-regulated genes PSA, KLK2, and TMPRSS2. Thus, we constructed the screening system for the specific androgen-dependent growth inhibitor as shown in Fig. 1G.

We screened 50,000 culture broths of microorganisms and found two broths that showed specific androgen-dependent growth inhibition in LNCaP prostate cancer cells. One of them contains androprostamines that have been previously reported by us [24,37] and the other contains DNT. DNT inhibited the AR-dependent transcriptional activity and AR nuclear translocation.

Androprostamine A repressed R1881-induced androgen-regulated gene expression, and dramatically inhibited R1881-induced prostate-specific antigen (PSA) levels. However, APA did not act as an AR antagonist, and did not inhibit AR transcriptional activity. Androprostamine A significantly inhibited the growth of VCaP cells in severe combined immunodeficient mice upon oral administration [24], however, DNT did not show anti-tumor effects in mice due to the short blood half-life (data not shown). Furthermore, the inhibitory effect of DNT on androgen-dependent growth in VCaP cells was one-tenth that of LNCaP cells (Fig. 2C). Because VCaP cells overexpress full-length AR and AR variants, and LNCaP cells express full-length AR only, differences in the effect of DNT on the androgen-dependent growth of prostate cancer cells may occur [9]. Although DNT inhibited the AR-dependent transcriptional activity in 22Rv1 cells because 22Rv1 cannot grow in response to androgen, we could not detect the inhibitory effect of DNT on the androgen-dependent growth in 22Rv1 cells by our screening system.

In conclusion, through this screening study, we identified that DNT specifically inhibited the androgen-dependent growth of human prostate cancer cells. DNT inhibited the AR-dependent transcriptional activity and AR nuclear translocation. Unfortunately, although DNT did not show an anti-tumor effect in mice due to the short half-life in blood, derivative research of DNT may overcome this challenge.

Prostate cancer is a long-term disease and progresses from androgen-dependent to CRPC and NEPC with various treatments. While the prognosis is good for patients diagnosed with localized disease, the prognosis remains poor for patients with advanced or metastatic prostate cancer.

This study proposed a new screening method for the detection of AR function inhibitors and identified DNT as a possible anti-prostate cancer agent.

## Data Availability

All relevant data are available from the corresponding author upon reasonable request.
